# TLR3 Activation of Hepatic Stellate Cell Line Suppresses HBV Replication in HepG2 Cells

**DOI:** 10.3389/fimmu.2018.02921

**Published:** 2018-12-17

**Authors:** Biao Zhang, Yu Liu, Xu Wang, Jieliang Li, Xiqiu Xu, Le Guo, Wen-Zhe Ho

**Affiliations:** ^1^School of Basic Medical Sciences, Wuhan University, Wuhan, China; ^2^Department of Pathology and Laboratory Medicine, Temple University Lewis Katz School of Medicine, Philadelphia, PA, United States

**Keywords:** hepatic stellate cells, hepatitis B virus, interferon-β, interferon-λ, Toll-like receptor 3, interferon-stimulated genes

## Abstract

There is limited information about the role of hepatic stellate cells (HSCs) in the liver innate immunity against hepatitis B virus (HBV) infection. We thus examined whether hepatic stellate cell line (LX-2) can be immunologically activated and produce antiviral factors that inhibit HBV replication in HepG2 cells. We found that LX-2 cells expressed the functional Toll-like receptor 3 (TLR3), activation of which by PolyI:C resulted in the selective induction of interferon-β (IFN-β) and IFN-λs, the phosphorylation of IFN regulatory factor 3 (IRF3) and IRF7. When HepG2 cells were treated with supernatant (SN) from PolyI:C-activated LX-2 cells, HBV replication was significantly inhibited. IFN-β and IFN-λ appeared to contribute to LX-2 SN-mediated HBV inhibition, as the antibodies to IFN-β and IFN-λ receptors could largely block the LX-2 SN action. Mechanistically, LX-2 SN treatment of the HepG2 cells induced a number of antiviral IFN-stimulated genes (ISGs: ISG20, ISG54, ISG56, OAS-1, Trim22, and Trim25) and facilitated the phosphorylation of STATs. These observations support further studies on the role of HSCs in the liver innate immunity against HBV infection.

## Introduction

Hepatitis B virus (HBV) infection is a frequent cause of severe liver diseases including liver cirrhosis and hepatocellular carcinoma (1). In infected hepatocytes, HBV DNA can integrate into host cell genome or present as non-integrated covalently closed circular DNA (cccDNA), which serves as a template for virus replication and persistent infection (2). Interferon alpha (IFN-α) and nucleos(t)ide analog-based inhibitors are the current treatment strategies for people infected with HBV (3). To establish and maintain persistent replication, HBV has evolved multiple strategies to evade the host innate and adaptive immune responses (4). Although there is controversy about whether HBV has a negative impact on host cell innate immunity, studies from different laboratories have shown that HBV compromises the immune system and establishes chronic infection. Lebosse et al. reported that the innate immune responses were impaired in untreated patients with chronic HBV infection (5). It has also been documented that the expression of IFN-β was suppressed by HBV infection in tupaia belangeri model (6). Besides, HBV can suppress TLR-mediated innate immune response in the liver cells (7). However, a recent study reported that HBV infection had little effect on signal pathway of pattern recognition receptors, and failed to induce IFN and IFN-stimulated genes in human hepatocytes (8). In addition, Suslov et al. showed that liver tissues from HBV-infected patients did not induce the innate immune response (9). These controversial studies underline the importance of our study which determines whether bystander cells such as stellate cells are involved in the liver innate immunity against HBV infection/replication.

Toll-like receptors (TLRs), the sensors of host cell innate immunity to invading pathogens, play an important role in recognizing pathogen-associated molecular patterns (PAMPs). Among identified human cell TLRs, TLR3 is a key sensor for viral infections, participating in virus-mediated innate immune responses (10, 11). A number of studies have demonstrated the expression of TLR3 in the liver cells, such as primary hepatocytes (12), Kupffer cells (13), hepatic dendritic cells (14). Importantly, TLR3-mediated antiviral function was confirmed by the observation that HBV replication could be inhibited by intravenous injection of the TLR3 ligand in a HBV transgenic mice model (15). The expression of type I IFNs induced by TLR3 ligand polyI:C reduced the cytoplasmic HBV pregenomic RNA (pgRNA)-containing capsids (16). In addition, TLR3 activation of Kupffer cells and sinusoidal endothelial cells potently inhibits HBV replication through production of IFN-β in mice (17).

Hepatic stellate cells (HSCs) are a key nonparenchymal component in the sinusoid compartment with multiple functions in the liver (18). In normal liver, HSCs represent 5–8% of the total number of liver cells (19) and maintain a non-proliferative, quiescent phenotype (20). The most characterized function of HSCs is the ability to transdifferentiate from a quiescent phenotype into highly proliferative, contractile, and wound-healing myofibroblast (21). The activated HSCs produce fibril-forming collagens and promote net deposition of fibrotic extracellular matrix to repair liver injury (21). In addition, accumulating evidence has shown that HSCs can function as antigen-presenting cells that present lipid antigens to natural killer T cells (22). HSCs also act as a regulatory bystander to facilitate differentiation and accumulation of regulatory T cells (Tregs) (23). We demonstrated that TLR3 signaling of a human stellate cell line (LX-2) could inhibit hepatitis C virus infection of hepatocytes (24). Here, we examined whether LX-2 cells have the ability to mount a TLR3 activation-mediated induction of antiviral factors in LX-2 cells and inhibit HBV replication in HepG2 cells.

## Materials and Methods

### Reagent

All culture reagents were purchased from GIBCO (Grand Island, NY, USA). All culture plasticware was obtained from Corning (Corning, NY, USA). LyoVec transfection reagent and polyinosinic-polycytidylic (PolyI:C) were purchased from InvivoGen (San Diego, CA, USA). IFN-β was purchased from Peprotech (Rocky Hill, NJ, USA). The pCMV ayw HBV proviral construct was kindly provided by Dr. Mingyu Lv (Jilin University, China). IFN-λ1 and neutralization antibodies against IFN-β, IL-28Rα, and IL-10Rβ were purchased from R&D (Minneapolis, MH, USA). TCI was obtained from EMD Millipore (Burlington, MA, USA).

### Cell Culture

Human hepatic stellate cell line (LX-2) and HepG2 cells were cultured in Dulbecco's modified Eagle medium (DMEM) supplemented with 10% fetal bovine serum, 100 IU/ml Penicillin and 100 μg/ml Streptomycin. Cells were cultured in an incubator at 37°C with 5% CO_2_ and 100% humidity. Cell culture medium was changed every 3 days.

### Transfection

LX-2 cells were seeded in a 48-well plate (10^4^/well) for 12 h. The cells were then transfected with PolyI:C by LyoVec transfection reagent for 4 h. LyoVec-treated cells were used as a vehicle control. The pCMV ayw HBV proviral construct (300 μg/ml) was transfected into HepG2 cells with LyoVec transfection reagent. The cell cultures was replaced with fresh culture medium 4 h post-transfection.

### RNA Extraction and Quantitative Real-Time PCR

Total RNA was extracted from cells with Tri-Reagent (Molecular Research Center, Cincinnati, OH, USA) according to the manufacturer's protocol. Total RNA (1 μg) was subjected to the reverse transcription using reagents purchased from Promega (Fitchburg, WI, USA). The quantitative real-time PCR was performed with iQ SYBR Green Supermix (Bio-Rad Laboratories, Hercules, CA, USA) as previously described (25).

### Western Blotting

Total cell lysates from LX-2 cells transfected with PolyI:C or from HepG2 cells treated with supernatant from LX-2 cells cultures were prepared by the cell extraction buffer (Thermo Fisher Scientific, Waltham, MA, USA) according to the manufacturer's instructions. The protein concentrations of cell lysates were measured with BCA Protein Assay Kit (Beyotime, Shanghai, China). Equal amount of protein lysates (30 μg) was separated on 4–12% Bis-Tris gels (Invitrogen, Waltham, MA, USA) and transfected to an Immunobiolon-P membrane (EMD Millipore, Burlington, MA, USA). The membranes (blots) were incubated with primary antibodies (1:1,000 dilution) in 5% non-fat milk in TBS with 0.1% Tween 20 (TBST) overnight at 4°C. Horseradish peroxidase-conjugated rabbit second antibodies were diluted at 1:2,000 to 1:8,000 in 2% non-fat milk TBST. Blots were developed with SuperSignal West Pico Chemiluminescent Substrate (Thermo Fisher Scientific, Waltham, MA, USA). All antibodies were obtained from Cell Signaling Technology (Cell Signaling Technology, Danvers, MA, USA). Densitometric analysis was performed with ImageJ 1.44 software.

### ELISA

IFN-β and IFN-λ1/3 protein levels in LX-2 culture SN were measured with ELISA kit (IFN-β: Invitrogen, Waltham, MA, USA; IFN-λ1/3: R&D system Inc., Minneapolis, MN, USA) according to the manufacturer's instructions. HBeAg and HBsAg in HepG2 cell cultures were measured with ELISA kit (Wantai Pharm, Beijing, China) according to the manufacturer's instructions.

### Data Analysis

Data were expressed as the mean ± standard deviation, representative of three independent experiments. Statistical significance was measured by Student's *t-*test or one-way analysis of variance followed by the Newman–Keul's test. Statistical significance was defined as ^*^p < 0.05, ^**^p < 0.01, or ^***^p < 0.001.

## Results

### TLR3 Activation of HSCs Induces IFN-β, IFN-λ, and Phosphorylation of IRF3 and IRF7

**Figure 1:**
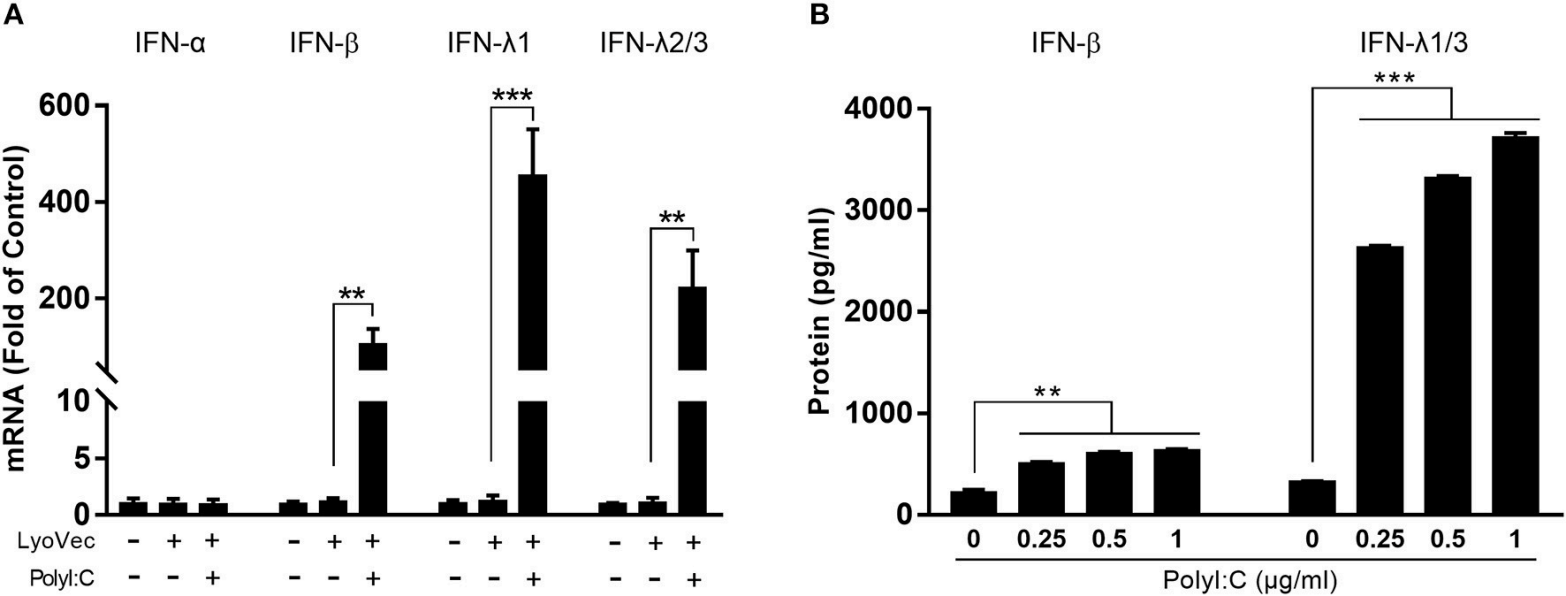
Effect of TLR3 activation on IFN-β and IFN-λ expression in LX-2 cells. **(A)** LX-2 cells were stimulated with PolyI:C (1 μg/ml) for 12 h. Total RNA extracted from cells was subjected to RT-qPCR for the mRNA levels of IFN-α, IFN-β, IFN-λ1, and IFN-λ2/3. **(B)** LX-2 cells were stimulated with different concentrations of PolyI:C (0.25, 0.5, and 1 μg/ml) for 12 h and cultured for 48 h post-stimulation. SN was collected for ELISA to measure the protein levels of IFN-β and IFN-λ1/3. The results are mean ± SD of three different experiments (**p < 0.01, ***p < 0.001).

**Figure 2:**
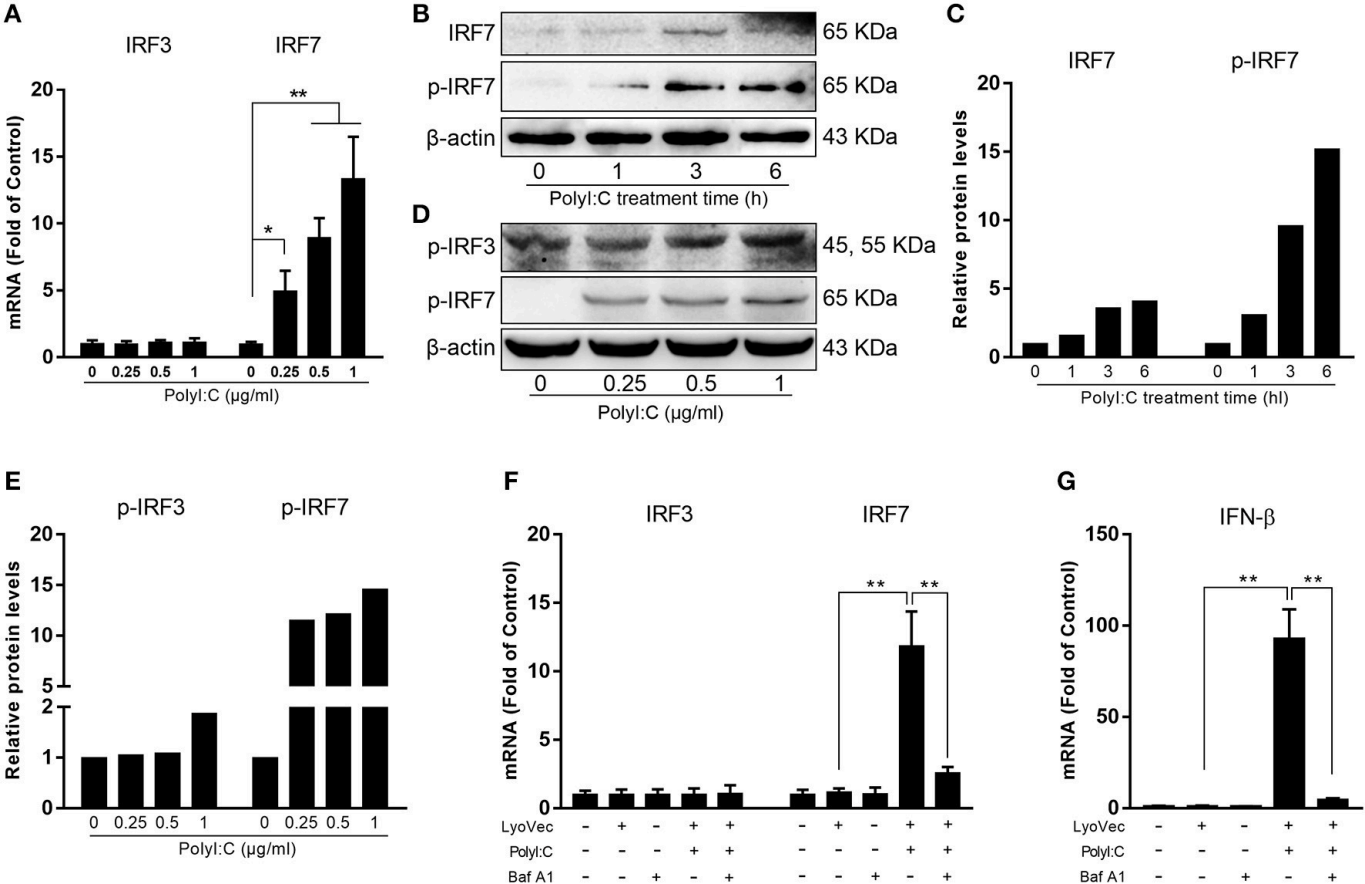
Effect of PolyI:C on the activation of IRF3 and IRF7 expression. **(A)** LX-2 cells were stimulated with PolyI:C (1 μg/ml) for 12 h. Total RNA extracted from cells was subjected to the RT-qPCR for the mRNA levels of IRF3 and IRF7. **(B,C)** LX-2 cells were stimulated with PolyI:C (1 μg/ml) for the indicated time period. Protein extracted from the cells were subjected to Western blotting for IRF7 and p-IRF7. **(D,E)** LX-2 cells were stimulated with PolyI:C (1 μg/ml) for 6 h. Protein extracted from the cells were subjected to Western blotting for p-IRF3 and p-IRF7. Densitometry analysis of the blot was performed with ImageJ 1.44 software. **(F,G)** LX-2 cells were pretreated with or without bafilomycin A1 (100 nM) for 1 h and then transfected with PolyI:C (1 μg/ml), total RNA extracted from cells was subjected to the RT-qPCR for the mRNA levels of IRF3, IRF7, and IFN-β. The results are mean ± SD of three different experiments (*p < 0.05, **p < 0.01).

We first examined whether PolyI:C could activate TLR3 in human hepatic stellate cells (LX-2). PolyI:C was added into the LX-2 cell cultures and we found it had little effect on TLR3 activation in LX-2 cells (Supplementary Figure 1). As shown in Figure 1, PolyI:C treatment of LX-2 cells induced IFN-β and IFN-λ expression at both mRNA and protein levels. The effect of PolyI:C on IFN-β and IFN-λ expression was dose-dependent (Figure 1). Because IRF3 and IRF7 have a key role in upregulation of type I IFNs, we examined the effect of PolyI:C on the expression of IRF3 and IRF7 in LX-2 cells. While PolyI:C could induce the expression of IRF7, it had little effect on IRF3 (Figure 2A). At protein level, PolyI:C significantly upregulated the phosphorylation of IRF7 (Figures 2B,C). Phosphorylation level of IRF3 and IRF7 were positively correlated with the concentrations of PolyI:C used to treat LX-2 cells (Figures 2D,E). To confirm the role of TLR3 in PolyI:C-stimulated IFN-β expression, LX-2 cells were pretreated with bafilomycin A1, a known inhibitor of TLR3 function, prior to PolyI:C stimulation. As shown in Figure 2F, TLR3 activation-mediated IRF expression was compromised by the pretreatment of LX-2 cells with bafilomycin A1. In addition, PolyI:C-mediated IFN-β expression was significantly inhibited by bafilomycin A1 pretreatment (Figure 2G). In addition, TCI, a TLR3/dsRNA complex inhibitor (26), also could significantly block the effect of PolyI:C on the induction of IFNs and IRF7 (Supplementary Figure 2). HBV containing SN from HepG2 cell cultures had little effect on IFN induction (Supplementary Figure 3).

### LX-2 SN Inhibits HBV Replication in HepG2 Cells

**Figure 3:**
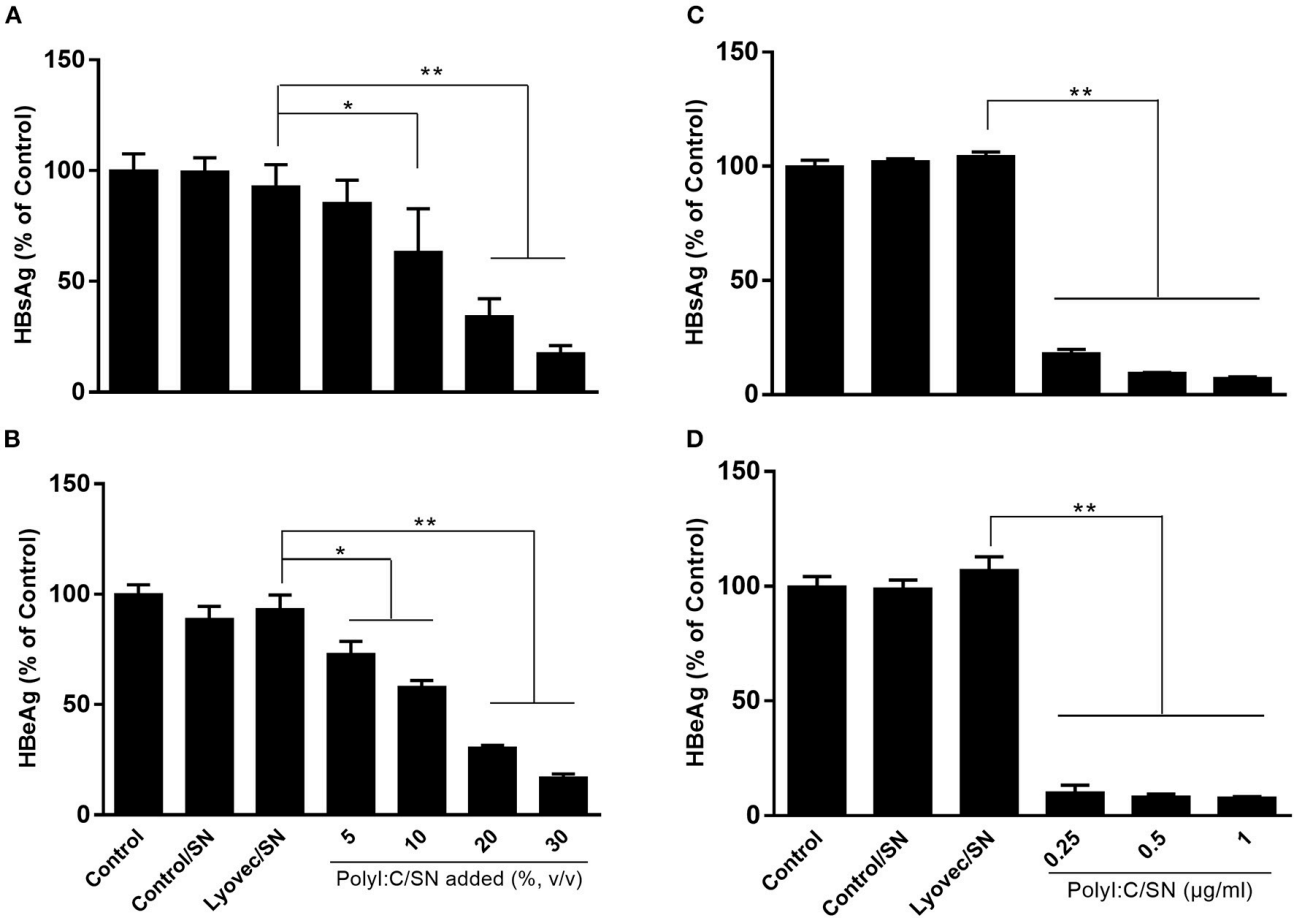
Effect of SN from activated LX-2 cultures on HBV replication in HepG2 cells. HepG2 cells were pretreated with SN from control (Control/SN), LyoVec (LyoVec/SN), or PolyI:C (PolyI:C/SN) -stimulated LX-2 cells cultures for 24 h. HepG2 cells transfected with HBV plasmid (300 ng/ml) were maintained in fresh medium for 48 h. **(A,B)** HepG2 cells were pretreated with indicated volumes of PolyI:C (1 μg/ml)-stimulated LX-2 cultures for 24 h. HBV antigen HBsAg **(A)** and HBeAg **(B)** in SN from HepG2 cultures were detected by ELISA. **(C,D)** HepG2 cells were pretreated with 20% (volume to volume ratio [v/v]) of indicated SNs collected from 0.25, 0.5, or 1 μg/ml PolyI:C-stimulated LX-2 cultures. HBV antigen HBsAg and HBeAg in SN from HepG2 cultures were detected by ELISA. The results are mean ± SD of three different experiments (*p < 0.05, **p < 0.01).

We then determined whether supernatant (SN) from PolyI:C-stimulated LX-2 cultures inhibits HBV replication in HepG2 cells. As shown in Figure 3, pretreatment of HepG2 cells with SN from PolyI:C-stimulated LX-2 cultures significantly inhibited the release of HBeAg and HBsAg from HepG2 cells. This inhibitory effect on the HBV antigen expression was dependent on the percentage of SN (from PolyI:C-stimulated LX-2 cultures) added to HepG2 cultures (Figures 3A,B) and the concentrations of PolyI:C used to stimulate LX-2 cells (Figures 3C,D). The transfection of HBV plasmid into HepG2 cells had little effect on TLR3 activation and IFN induction (Supplementary Figure 4).

### IFN-β and IFN-λ Are the Key Factors in LX-2 SN-Mediated HBV Inhibition

**Figure 4:**
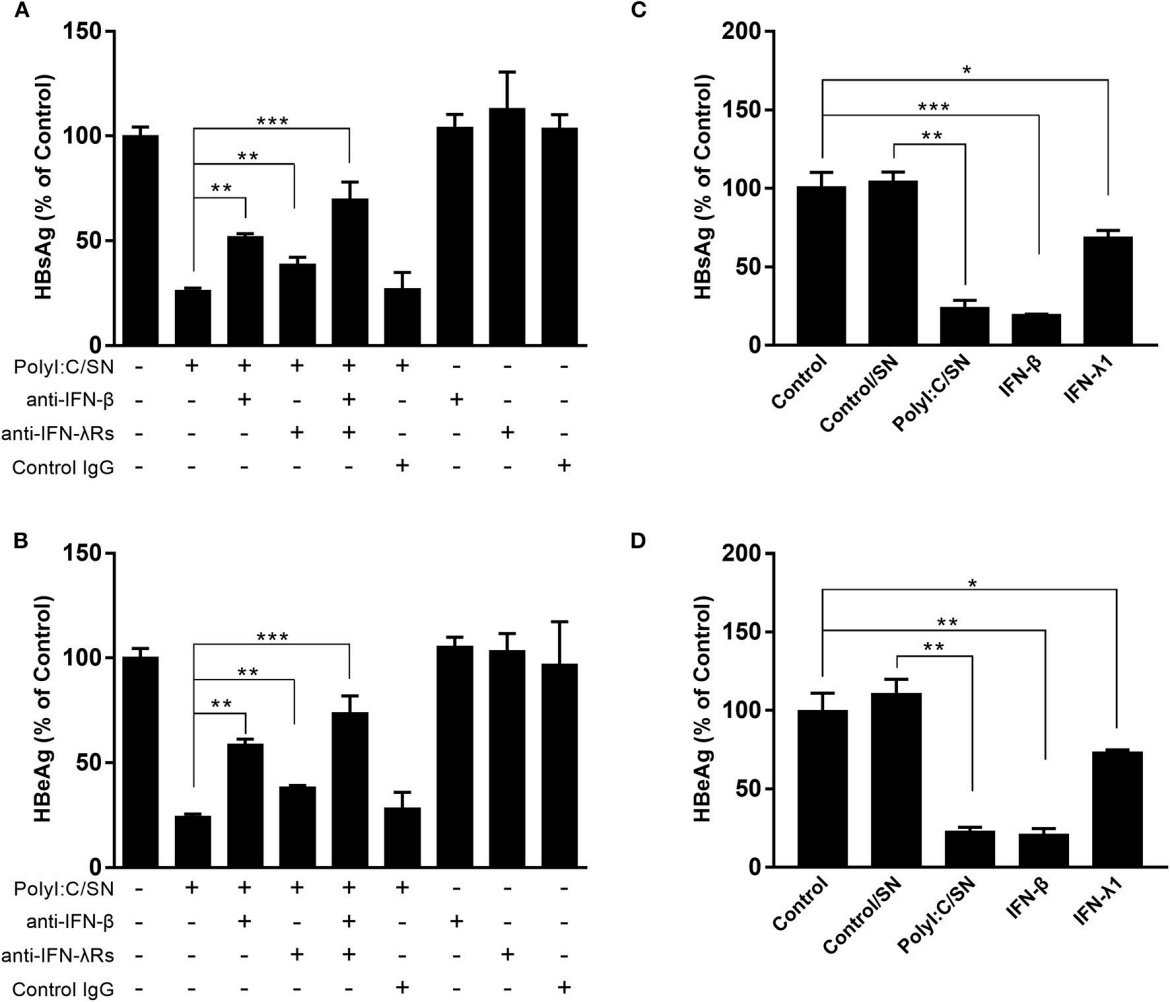
Effect of the antibodies to IFN-β or IFN-λ receptors on LX-2 supernatant (SN)-mediated HBV inhibition. **(A,B)** PolyI:C-stimulated LX-2 culture SN was preincubated with anti-IFN-β neutralization antibody (10 μg/ml) for 1 h and then used to treat HepG2 cells for 24 h prior to HBV plasmid transfection. For IFN-λ receptors pretreatment, the neutralization antibodies against IL-28Rα (25 μg/ml) and IL-10Rβ (10 μg/ml) was added to HepG2 cell cultures for 1 h prior to the addition of LX-2 SN. HBsAg and HBeAg in the culture SN were measured by ELISA 48 h after HBV plasmid transfection. **(C,D)** HepG2 cells were pretreated with exogenous IFN-β (1 ng/ml), IFN-λ (4 ng/ml), and SN from control (Control/SN) or PolyI:C (PolyI:C/SN) -stimulated LX-2 cells cultures for 24 h. HepG2 cells transfected with HBV plasmid (300 ng/ml) were maintained in cultures for 48 h. SN was collected 48 h post-transfection, and HBsAg and HBeAg were measured by ELISA. The results are mean ± SD of three different experiments (*p < 0.05, **p < 0.01, ***p < 0.001).

As shown in Figure 1, the TLR3 activation of LX-2 cells induces the expression of both IFN-β and IFN-λ at mRNA and protein levels. To determine the roles of IFNs in LX-2 cells-mediated anti-HBV activity, we used the neutralization antibody to IFN-β to treat the LX-2 SN or antibodies to IFN-λ receptors to treat HepG2 cells. As shown in Figures 4A,B, the antibody to IFN-β could largely block the inhibitory effect of SN from PolyI:C-stimulated LX-2 cultures on HBV replication. In addition, the pretreatment of HepG2 cells with the antibodies to IFN-λ receptors (IL-28Rα and IL-10Rβ) also compromised LX-2 SN-mediated HBV suppression, although the extent of the antibodies effect was less than the antibody to IFN-β. As shown in Figures 4C,D, IFN-β, IFN-λ1, and SN from PolyI:C-stimulated LX-2 cultures all significantly inhibited the replication of HBV in HepG2 cells. IFN-β was the most potent inhibitor among them.

### LX-2 SN Induces ISGs in HepG2 Cells

**Figure 5:**
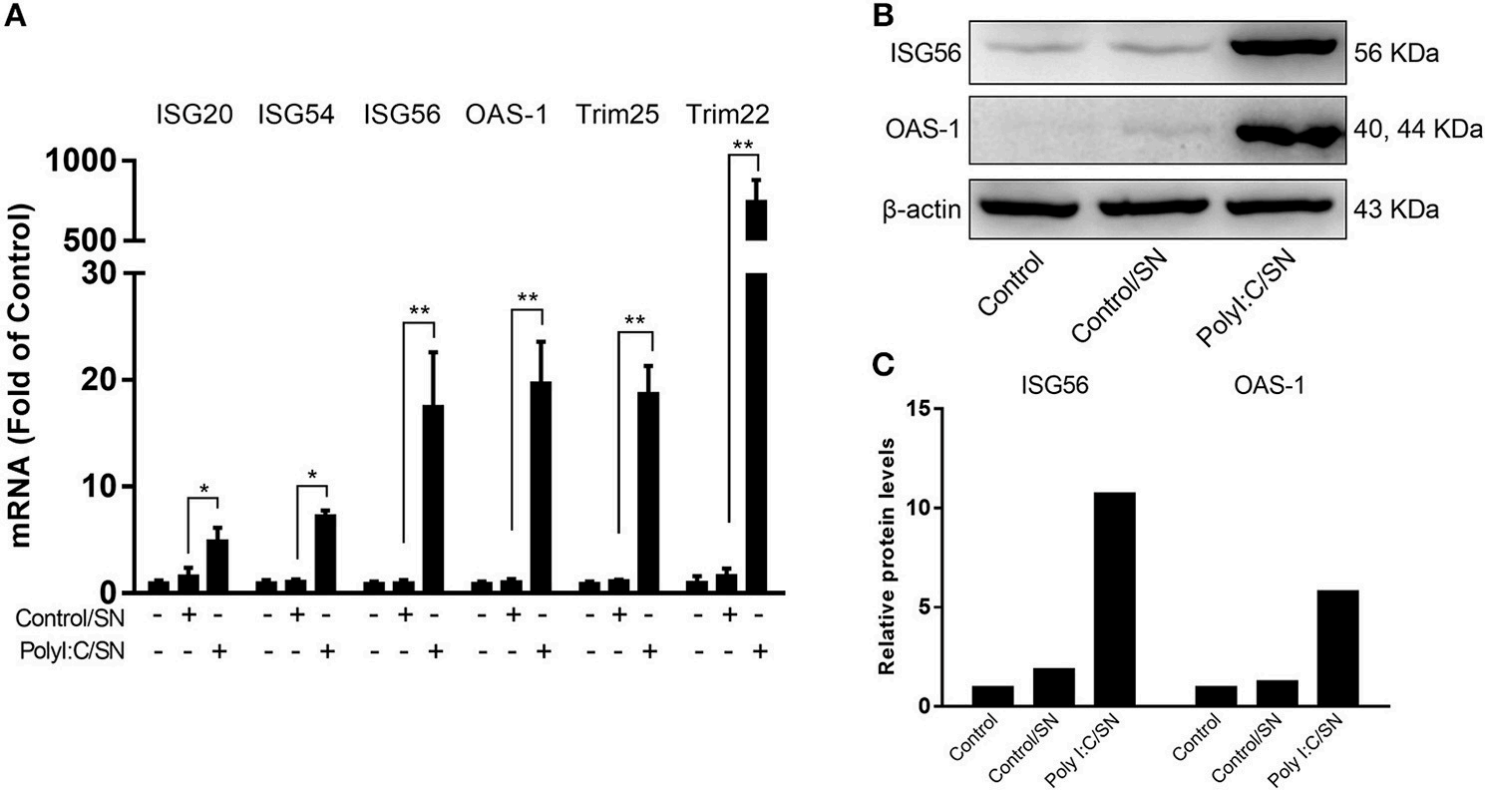
Effect of PolyI:C-stimulated LX-2 culture SN on ISGs expression in HepG2 cells. **(A)** LX-2 cells were stimulated with PolyI:C (1 μg/ml) for 12 h and further incubated to 48 h post-stimulation. Culture SN was collected for treatment of HepG2 cells (20% v/v) for 12 h. RNA was extracted for the expression of the indicated ISGs by RT-qPCR. The results are mean ± SD of three different experiments (^*^p < 0.05, ^**^p < 0.01). **(B,C)** HepG2 cells were treated with PolyI:C-stimulated LX-2 culture SN for 24 h and proteins extracted from the cells were subjected to Western blotting for ISG56, OAS-1, and β-actin. Densitometry analysis of the blot was performed with ImageJ 1.44 software.

To determine whether LX-2 SN can induce ISGs in the hepatocytes, we next examined the effect of LX-2 SN on the ISGs expression in HepG2 cells. The mRNAs of ISGs, including ISG20, ISG54, ISG56, OAS-1, Trim25, and Trim22, were significantly upregulated in HepG2 cells by SN from PolyI:C-stimulated LX-2 cultures (Figure 5A). In addition, SN from TLR3-activated LX-2 cultures induced the expression of ISG56 and OAS-1 at protein level in HepG2 cells (Figures 5B,C).

### LX-2 SN Activates STAT Signal Pathway

**Figure 6:**
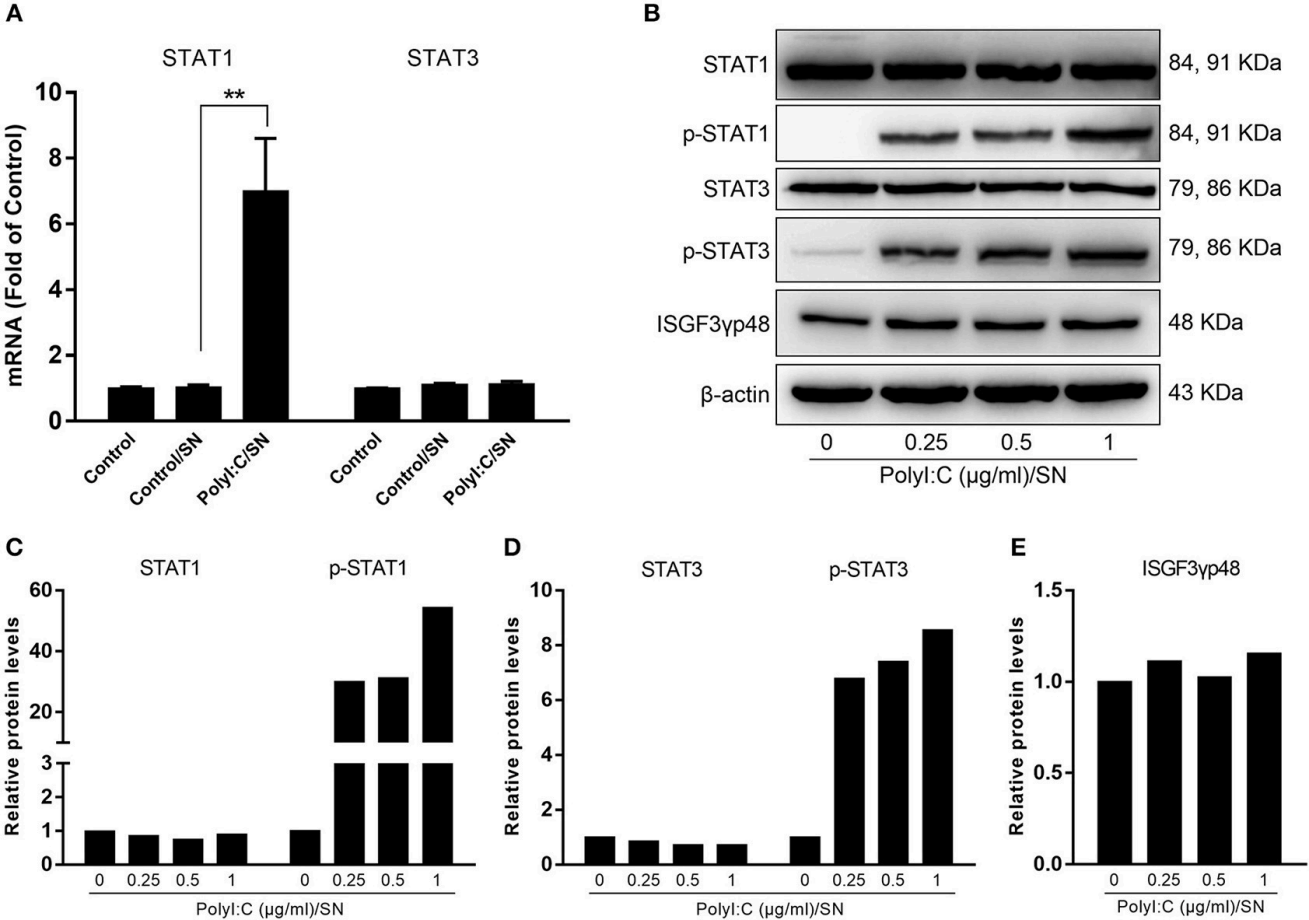
Effect of PolyI:C-stimulated LX-2 culture SN on the activation of STATs in HepG2 cells. LX-2 cells were stimulated with 0.25, 0.5, and 1 μg/ml PolyI:C for 12 h, then washed 3 times, and further incubated in fresh medium to 48 h poststimulation. **(A)** SN from LX-2 cells stimulated with 1 μg/ml PolyI:C was collected to treat HepG2 cells for 12 h (20% v/v). Total RNA extracted from cells was subjected to the RT-qPCR for the mRNA levels of STAT1 and STAT3. The results are mean ± SD of three different experiments (**p < 0.01). **(B–E)** SN from LX-2 cells stimulated with indicated concentrations of PolyI:C was collected to treat HepG2 cells for 1 h (20% v/v). Proteins extracted from the cells were subjected to Western blotting for STAT1, p-STAT1, STAT3, p-STAT3, ISGF3γp48, and β-actin. Densitometry analysis of the blot was performed with ImageJ 1.44 software.

Because STAT1 and STAT3 are known to be the important regulators of ISGs driven by IFN-β and IFN-λ, we also examined the expression of STAT1 and STAT3 in LX-2 SN-treated hepatocytes. As shown in Figure 6A, LX-2 SN induced the mRNA expression of STAT1 but not STAT3. However, LX-2 SN could facilitate the phosphorylation of both STAT1 and STAT3 at protein level in a dose-dependent manner (Figures 6B–E).

## Discussion

**Figure 7:**
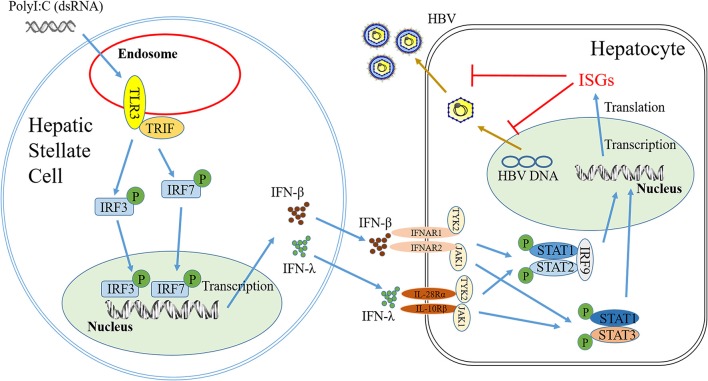
Hypothetical model of anti-HBV mechanism of TLR3 signaling of HSCs. Stimulation of HSCs with PolyI:C (dsRNA) activates TLR3 signal pathway, which facilitates phosphorylation and translocation of IRF3 and IRF7, initiating the production of IFN-β and IFN-λ. IFN-β and IFN-λ released from HSCs could binds to their corresponding receptors in hepatocytes and activate STAT signal pathway, inducing anti-HBV IFN-stimulate genes (ISGs), which can inhibit HBV replication in hepatocytes.

The interactions between HBV infection and liver innate immunity have a crucial role in the development of HBV disease. It is well-known that HBV has the ability to establish persistent infection in liver by evading and/or suppressing the host cell innate immunity (27, 28). HBV infection had little effect on host cellular gene expression and induction of IFNs (29) and the ISGs (30). Therefore, to immunologically activate the liver cell-mediated innate immunity suppressed by HBV is of a great interest. In this study, we attempted to determine whether hepatic stellate cells, a non-HBV target in liver, are involved in the innate immunity against HBV infection. We showed that the SN from immunologically activated LX-2 cell cultures potently induced the expression of the antiviral ISGs in HepG2 cells, including ISG20, ISG54, ISG56, OAS-1, Trim25, and Trim22 (Figure 5). These antiviral ISGs inhibit HBV replication at different stages of viral life cycle (Figure 7). For example, ISG20 has the ability to inhibit HBV replication through degradation of HBV RNA (31), TRIM22 suppresses HBV core promoter activity, thus inhibiting HBV gene expression and viral replication in both vitro and vivo systems (32). ISG54 and ISG56 contribute significantly to the inhibition of HBV replication at both transcriptional and posttranscriptional steps (33). In addition, we found that SN from PolyI:C-stimulated LX-2 cells could facilitate phosphorylation of STAT1 and STAT3 (Figures 6B–E), which is likely to be responsible for LX-2 cell SN-mediated the induction of the antiviral ISGs in HepG2 cells.

As the sensors of host cell-mediated innate immunity, TLRs are involved in the immune defense against viral infections, including HBV (34). HBV, however, could counteract to the innate immune response stimulated by TLR3 activation of hepatocytes (7). The inhibitory effect by HBV is at least partially mediated through suppression of IFN-β and the ISGs (7). We demonstrated that TLR3 activation of LX-2 cells resulted in the induction of the multiple antiviral factors that have the ability to inhibit HBV replication in HepG2 cells. Our investigation into the mechanism for the induction n of IFN-β and IFN-λ showed that TLR3 activation of LX-2 cells facilitated the phosphorylation of IRF3 and IRF7 (Figures 2B–E), the key positive regulators of IFN signaling pathway. In addition to TLR3, the cytosolic RNA helicases retinoic acid-inducible I (RIG-I) and melanoma differentiation-associated gene 5 (MDA5) also can recognize the dsRNA mimic. To determine which signal pathway plays the major role in PolyI:C-stimulated IFN-β and IFN-λ induction in LX-2 cells, we used the bafilomycin A1, an inhibitor of TLR3 signal pathway, to treat LX-2 cells prior to PolyI:C stimulation. The vacuolar H+ATPases (V-ATPases) are a family of ATP-driven proton pumps responsible for the acidification of a variety of intracellular compartments. V-ATPases provide the acidic environment required for dissociation of internalized ligand-receptor complexes within endosomes (35). TLR3-mediated signaling is initiated from endosomal compartments in cells, requiring endosomal acidification (36). Bafilomycin A1, as a vaculaor H+ATPase inhibitor, can effectively block endosome acidification to inhibit TLR3 signaling. We observed that bafilomycin A1 could suppress the induction of IFN-β and IRF-7 by PolyI:C, indicating that TLR3 is the key sensor responsible for PolyI:C- mediated the induction of IFN-β in LX-2 cells (Figures 2F,G). Also, the pretreatment of LX-2 cells with TCI significantly block the induction of IFNs and IRF7 by PolyI:C (Supplementary Figure 2).

Because the neutralization antibody to IFN-β could largely block the anti-HBV effect by SN from PolyI:C-activated LX-2 cell cultures, IFN-β is likely to be a major player responsible for the anti-HBV effect. In addition, we observed that the antibodies to IFN-λ receptors could partially block LX-2 SN-mediated HBV inhibition, suggesting that IFN-λ also contributes to the LX-2 SN actions. The role of IFN-β and IFN-λ in LX-2 SN-mediated HBV inhibition was further confirmed by the observation that when added to HBV-infected HepG2 cell cultures, recombinant IFN-β or IFN-λ significantly inhibited the HBV replication (Figures 4C,D). These findings are consistent with other studies showing that IFN-β could suppress HBV replication in HepG2 cells and HBV-infected chimeric mice (17, 37, 38). In our previous study, we found that IFN-λ from polyI:C-stimulated LX-2 SN contributed to HCV inhibition in Huh7 cells (24). Several early studies also showed that IFN-λ could inhibit HBV replication in HepG2 cells and HBV-Met cells, a hepatocyte cell line derived from HBV-transgenic mice (39, 40). Recently, Xu et al reported that IFN-λ could inhibit HBV replication in HepG2 cells by inducing the expression of CBFβ (41).

In summary, we provide the experimental evidence to support the notion that the TLR3 signaling of LX-2 cells is beneficial for the immunological control of HBV replication in HepG2 cells. However, future investigations with human liver tissues from people infected with HBV are necessary in order to prove that hepatic stellate cells indeed participate in liver innate immunity against HBV infection. These future studies will determine whether HSCs are a potentially crucial and alternative target for immunological intervention to control HBV infection in liver.

## Author Contributions

BZ, W-ZH, XW, and JL conceived of and designed the experiments. BZ, YL, XX, and LG performed the experiments. BZ, XW, and W-ZH analyzed the data. BZ made the figures. W-ZH contributed reagents, materials, and analysis tools. BZ and W-ZH wrote the manuscript. All authors reviewed the manuscript.

### Conflict of Interest Statement

The authors declare that the research was conducted in the absence of any commercial or financial relationships that could be construed as a potential conflict of interest.
