# Supplementary material for: TLR3 Activation of Hepatic Stellate Cell Line Suppresses HBV Replication in HepG2 Cells
Source: Front Immunol. 2018 Dec 17;9:2921. doi: 10.3389/fimmu.2018.02921 (PMC6304368; doi:10.3389/fimmu.2018.02921)
Supplement: Supplementary file 1 [file Data_Sheet_1.pdf]

TLR3 Activation of Hepatic Stellate Cell Line Suppresses HBV Replication in HepG2 Cells

Supplementary Figures

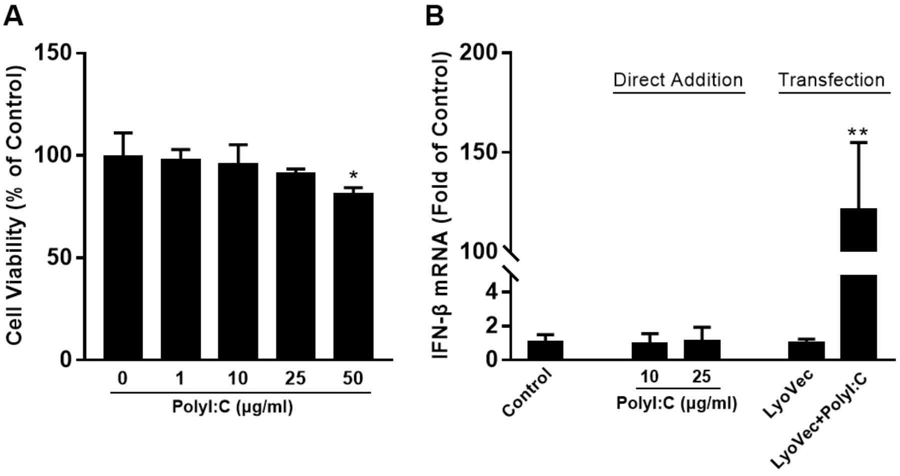

Supplementary Figure 1. Effect of PolyI:C treatment on cell viability and IFN-β mRNA expression. (A) The cytotoxicity effect of PolyI:C treatment on LX-2 cells. LX-2 cells were treated with/without PolyI:C at the indicated concentrations for 48 h. The cell viability was analyzed by MTS assay. Data are showed as the absorbance (490 nm) relative to untreated control, which is defined as 100%. (B) LX-2 cells were treated with the indicated concentrations of PolyI:C either directly or by transfection for 12 h. Total RNA extracted from cells was subjected to RT-qPCR for the mRNA levels of IFN-β. The results are mean ± SD of three different experiments (**p* < 0.05, ***p* < 0.01).

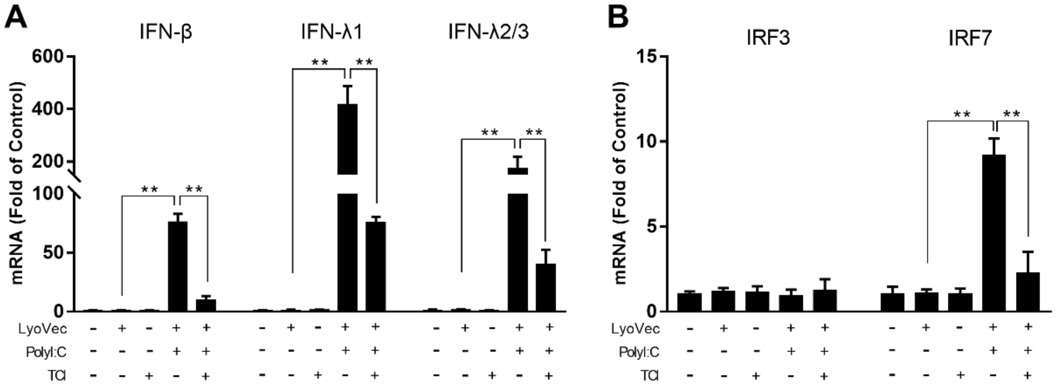

Supplementary Figure 2. TCI treatment inhibited the expression of IFNs and IRFs. LX-2 cells were pretreated with/without TCI (100nM) for 1 h prior to PolyI:C activation. Total RNA extracted from the cells was subjected to the RT-qPCR for the

IFNs (A) and IRFs (B). The results are mean ± SD of three different experiments (** $p < 0.01$).

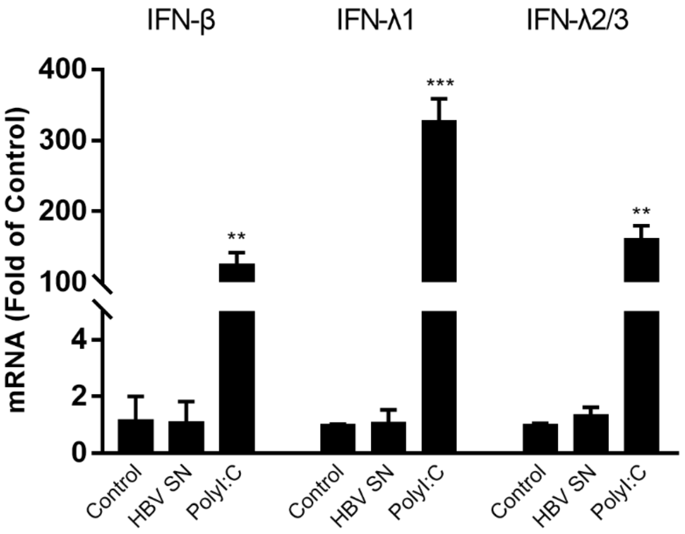

Supplementary Figure 3. The effect of HBV SN on TLR3 signal activation in LX-2 cells. HepG2 cells were transfected with HBV plasmid (300 ng/ml) for 4 h, and the SN was collected after 48 h after transfection. LX-2 cells were treated with HBV SN (20% v/v) or transfected with PolyI:C (1 μg/ml) for 12 h. Total RNA extracted from cells was subjected to RT-qPCR for IFNs mRNA**.** The results are mean ± SD of three different experiments (** $p < 0.01$, *** $p < 0.001$).

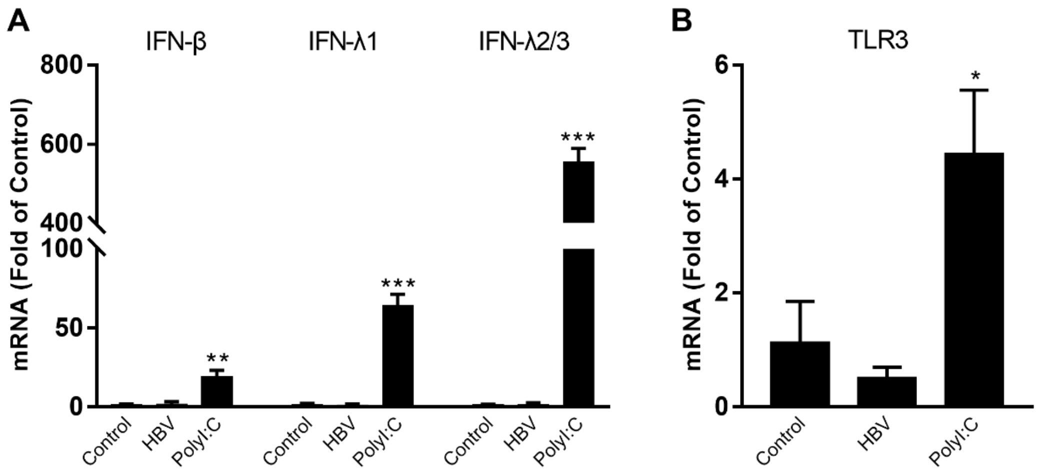

Supplementary Figure 4. The effect of HBV on TLR3 activation in HepG2 cells. HepG2 cells were transfected with HBV plasmid (300 ng/ml) or PolyI:C (1 μg/ml) for 12 h. Total RNA extracted from cells was subjected to RT-qPCR for IFNs mRNA (A)

and TLR3 (B). The results are mean ± SD of three different experiments (*$p < 0.05$, **$p < 0.01$, ***$p < 0.001$).
